# Supplementary material for: Content-rich biological network constructed by mining PubMed abstracts
Source: BMC Bioinformatics. 2004 Oct 8;5:147. doi: 10.1186/1471-2105-5-147 (PMC528731; doi:10.1186/1471-2105-5-147)
Supplement: Additional File 2 — The original results of the above study (non-essential files are deleted to keep the file size under the limit set by BMC bioinformatics). [file 1471-2105-5-147-S2.bz2 › chilibotAdditionalFile2/dip30/657ID10353244E11048/html/KPNB1_KPNA2.html]

 


 **KPNB1** and **KPNA2** 
  
Found 172 abstracts in PubMed, retrieved 30.  
 

 What does Google say? 
 PDF only 
| .edu only 

---

**Interactive relationship** (e.g. stimulation, inhibition, etc)

**Neutral relationship**- While performing a yeast two hybrid library screen to uncover novel PP2A interacting proteins, we discovered a specific interaction between a member of the importin beta karyopherin  [ **KPNA2** ]   [ **KPNB1** ]  beta superfamily, importin 9, and the A subunit of PP2A PR65.  Ref: 12670497 Biochem Biophys Res Commun, 2003
- Genetic and physical interactions between Srp1p and VDE imply direct involvement of karyopherin  [ **KPNA2** ]   [ **KPNB1** ]  mediated nuclear transport in this process.  Ref: 12588991 Mol Cell Biol, 2003
- The Saccharomyces cerevisiae NPC is comprised of 30 nucleoporins Nups, 13 of which contain phenylalanine glycine repeats FG Nups that bind karyopherins and facilitate the transport of karyopherin  [ **KPNA2** ]   [ **KPNB1** ]  cargo complexes.  Ref: 12604785 Proc Natl Acad Sci U S A, 2003
- We also show that both L and D amino acid containing peptides bind well to karyopherin  [ **KPNA2** ]   [ **KPNB1** ]  alpha 2.  Ref: 12504098 Biochem Biophys Res Commun, 2003
- We demonstrate that Nap1p can bind directly to Kap114p, the primary karyopherin  [ **KPNA2** ]   [ **KPNB1** ]  importin responsible for the nuclear import of H2A and H2B.  Ref: 12456659 EMBO J, 2002
- ... the L amino acid peptides bind more efficiently than the D amino acid peptide to karyopherin  [ **KPNA2** ]   [ **KPNB1** ]  alpha1.  Ref: 12504098 Biochem Biophys Res Commun, 2003
- An intracellular targeted NLS peptide inhibitor of karyopherin  [ **KPNA2** ]   [ **KPNB1** ]  alpha NF kappa B interactions.  Ref: 12504098 Biochem Biophys Res Commun, 2003
- A subgroup of the karyopherin  [ **KPNA2** ]   [ **KPNB1** ]  beta also called importin beta protein that includes budding yeast Kap104 and human transportin karyopherin  [ **KPNA2** ]   [ **KPNB1** ]  beta2 is reported to function as a receptor for the transport of mRNA binding proteins into the nucleus.  Ref: 12524331 Genetics, 2002

**Non-interactive relationship** (e.g. studied together, co-existance, homology, etc.)

- Prospective mimetics with linkers of, 10, or 12 atoms were synthesized and compared in a competition assay for binding to the nuclear import receptor subunit karyopherin  [ **KPNA2** ]   [ **KPNB1** ]  alpha, showing the 10 atom linker to be best and shorter ones ineffective.  Ref: 12841749 Org Lett, 2003
- We report here that the karyopherin  [ **KPNA2** ]   [ **KPNB1** ]  Mtr10p is required for the normal accumulation of mature Tlc1 and its proper localization to the nucleus.  Ref: 12167699 Mol Cell Biol, 2002
- Treatment with the karyopherin  [ **KPNA2** ]   [ **KPNB1** ]  specific inhibitor leptomycin B was toxic to T. brucei and eliminated the cytoplasmic SL RNA, suggesting that cytoplasmic SL RNA was dependent on the nuclear exporter exportin 1 XPO1.  Ref: 12684371 Eukaryot Cell, 2003
- In the present work, we have characterized the function of the karyopherin  [ **KPNA2** ]   [ **KPNB1** ]  Msn5p in the control of the cell cycle of Saccharomyces cerevisiae.  Ref: 12697814 Mol Cell Biol, 2003
- Karyopherin  [ **KPNA2** ]   [ **KPNB1** ]  alpha2 a control step of glucose sensitive gene expression in hepatic cells.  Ref: 11988093 Biochem J, 2002
- Most nucleocytoplasmic traffic through the nuclear pore complex is mediated by soluble receptors of the importin exportin or karyopherin  [ **KPNA2** ]   [ **KPNB1** ]  family.  Ref: 12637516 J Biol Chem< MedlineTA>J Biol Chem, 2002
- Furthermore, we demonstrate that Nap1p promotes the association of the H2A and H2B NLSs specifically with the karyopherin  [ **KPNA2** ]   [ **KPNB1** ]  Kap114p.  Ref: 12456659 EMBO J, 2002
- In the present study, by interaction with the GLUT2 loop, we have cloned the rat karyopherin  [ **KPNA2** ]   [ **KPNB1** ]  alpha2, a receptor involved in nuclear import.  Ref: 11988093 Biochem J, 2002
- The transitory tethering of karyopherin  [ **KPNA2** ]   [ **KPNB1** ]  alpha2 to GLUT2 at the plasma membrane might indicate that the receptor can load the cargo to be imported locally.  Ref: 11988093 Biochem J, 2002
- The amount of inactive karyopherin  [ **KPNA2** ]   [ **KPNB1** ]  alpha2 receptor reduced, in a dose dependent manner, the GLUT2 and liver pyruvate kinase mRNA levels by competition with endogenous active receptor.  Ref: 11988093 Biochem J, 2002
- A role for karyopherin  [ **KPNA2** ]   [ **KPNB1** ]  alpha2 in the transcription of two glucose sensitive genes was investigated by transfection of native and inactive green fluorescent protein karyopherin  [ **KPNA2** ]   [ **KPNB1** ]  alpha2 in GLUT2 expressing hepatoma cells.  Ref: 11988093 Biochem J, 2002
- We show that the yeast karyopherin  [ **KPNA2** ]   [ **KPNB1** ]  Kap121p functions in the targeting and assembly of the nucleoporin Nup53p into NPCs by recognizing a nuclear localization signal NLS in Nup53p.  Ref: 12403813 J Cell Biol, 2002
- The NLS of SXR is characterized as the bipartite type, and is recognized by the three molecular species of importin alpha Rch1  [ **KPNA2** ]  PTAC58  [ **KPNA2** ] , NPI1, and Qip1, in the presence of PTAC97  [ **KPNB1** ]  of importin beta to target the nuclear pore.  Ref: 12606758 Mol Pharmacol, 2003
- In either case, the Ran.GTP is then transported to the cytoplasm by the karyopherin  [ **KPNA2** ]   [ **KPNB1** ] , where it is hydrolyzed to Ran.GDP.  Ref: 11911364 RNA, 2002
- Furthermore, the transport defective 31 allele of the karyopherin  [ **KPNA2** ]   [ **KPNB1** ]  SRP1, but not the transport competent 49 allele, exhibited a tab phenotype.  Ref: 11914130 BMC Genet, 2002
- In nonpreferred nitrogen or nitrogen starvation, Gln3p is dephosphorylated and imported into the nucleus via karyopherin  [ **KPNA2** ]   [ **KPNB1** ]  alpha Srp1p.  Ref: 12624103 J Biol Chem, 2003
- Consistent with the hypothesis that Crm1 is not the only karyopherin  [ **KPNA2** ]   [ **KPNB1** ]  cofactor for MS2 Ran mediated mRNA export, we show that not only Crm1 but also CAS, transportin, importin beta and exportin t can all export mRNA from the nucleus when tethered via the MS2 RNA binding domain.  Ref: 11911364 RNA, 2002
- The present study suggests that, in concert with glucose metabolism, karyopherin  [ **KPNA2** ]   [ **KPNB1** ]  alpha2 transmits a signal to the nucleus to regulate glucose sensitive gene expression.  Ref: 11988093 Biochem J, 2002
- Reconstitution assays revealed that only precursor complexes are targeted to the nuclear envelope NE by karyopherin  [ **KPNA2** ]   [ **KPNB1** ]  alphabeta.  Ref: 11922673 J Mol Biol, 2002
- Results from this study showed that fertilized porcine embryos injected with double stranded RNA dsRNA corresponding to the importin alpha homologue karyopherin  [ **KPNA2** ]   [ **KPNB1** ]  alpha3 had significantly fewer nuclei following four days of culture than did embryos injected with dsRNA for another importin alpha homologue, karyopherin  [ **KPNA2** ]   [ **KPNB1** ]  alpha2, or two control groups.  Ref: 12548662 Mol Reprod Dev, 2003
- Karyopherin  [ **KPNA2** ]   [ **KPNB1** ]  mediated nuclear import of the homing endonuclease VMA1 derived endonuclease is required for self propagation of the coding region.  Ref: 12588991 Mol Cell Biol, 2003
- RNA export mediated by the minihelix motif is Ran dependent, which indicates the involvement of a karyopherin  [ **KPNA2** ]   [ **KPNB1** ]  related factor exportin that remained to be determined.  Ref: 12509441 J Biol Chem, 2003
- In contrast, the overexpression of karyopherin  [ **KPNA2** ]   [ **KPNB1** ]  alpha2 did not significantly stimulate GLUT2 and liver pyruvate kinase mRNA accumulation in green fluorescent protein sorted cells.  Ref: 11988093 Biochem J, 2002
- We found that HPV16 E6 interacted with the karyopherin  [ **KPNA2** ]   [ **KPNB1** ]  Kap alpha2 adapter and could enter the nucleus via a classical Kap alpha2beta1 mediated pathway.  Ref: 12551970 J Virol, 2003
- We report the development of a simple and highly efficient lipofection method for primary embryonic rat hippocampal neurons up to 25% transfection that exploits the M9 sequence of the non classical nuclear localization signal of heterogeneous nuclear ribonucleoprotein A1 for targeting beta 2 karyopherin  [ **KPNA2** ]   [ **KPNB1** ]  transportin 1.  Ref: 12044466 Neuroscience, 2002
- Kap123p is a yeast beta karyopherin  [ **KPNA2** ]   [ **KPNB1** ]  that imports ribosomal proteins into the nucleus prior to their assembly into preribosomal particles.  Ref: 12612077 Mol Cell Biol, 2003
- Protein cargoes that contain a classical nuclear localization signal NLS are transported into the nucleus through binding to a heterodimeric receptor comprised of importin karyopherin  [ **KPNA2** ]   [ **KPNB1** ]  alpha and beta.  Ref: 12672802 J Biol Chem< MedlineTA>J Biol Chem, 2003
- Karyopherin  [ **KPNA2** ]  beta1  [ **KPNB1** ]  levels do not change with age and present very little variation among donors.  Ref: 12051719 Biochem Biophys Res Commun, 2002
- Our results indicate that VIP1 is imported into the plant cell nucleus via the karyopherin  [ **KPNA2** ]   [ **KPNB1** ]  alphadependent pathway.  Ref: 12124400 Proc Natl Acad Sci U S A, 2002
- The Ran protein regulates nucleocytoplasmic transport mediated by the karyopherin  [ **KPNA2** ]   [ **KPNB1** ]  family of nuclear transport factors.  Ref: 11911364 RNA, 2002
- This karyopherin  [ **KPNA2** ]   [ **KPNB1** ]  mediated function can also be performed by the Kap95p Kap60p complex if the Kap121p binding domain of Nup53p is replaced by a classical NLS, suggesting a more general role for karyopherins in NPC assembly.  Ref: 12403813 J Cell Biol, 2002
- The specificity of the binding was restricted to GLUT2, and not GLUT1 or GLUT4, and to karyopherin  [ **KPNA2** ]   [ **KPNB1** ]  alpha2, not alpha1.  Ref: 11988093 Biochem J, 2002
- Second, the import of neoglycoproteins was not affected by microinjection of anti nuclear import factor importin karyopherin  [ **KPNA2** ]   [ **KPNB1** ]  beta antibodies while the NLS dependent transport was completely abolished.  Ref: 12672698 Glycobiology< MedlineTA>Glycobiology, 2002
- By using the srp1 49 mutant which is deficient in nuclear import of cargos with classical nuclear localization sequences cNLS, we show that proteasome precursor complexes associate with importin karyopherin  [ **KPNA2** ]   [ **KPNB1** ]  alphabeta, the cNLS receptor,.  Ref: 11922673 J Mol Biol, 2002
- Age dependent decreases in the protein concentrations of the nucleocytoplasmic transport factors karyopherin  [ **KPNA2** ]   [ **KPNB1** ]  alpha2, CAS, and RanBP1 were found by comparing fibroblast cultures obtained from young, mature, and old human donors.  Ref: 12051719 Biochem Biophys Res Commun, 2002
- All non coding RNAs are now known to depend on members of the karyopherin  [ **KPNA2** ]   [ **KPNB1** ]  family of Ran dependent nucleocytoplasmic transport factors for their nuclear export.  Ref: 12538759 J Cell Sci, 2003
- Proteins that contain a classical nuclear localization signal NLS are recognized in the cytoplasm by a heterodimeric import receptor composed of importin karyopherin  [ **KPNA2** ]   [ **KPNB1** ]  alpha and beta.  Ref: 12486120 J Biol Chem, 2003
- We have now developed a homogeneous solution based assay and show that NF kappa B interacts with karyopherin  [ **KPNA2** ]   [ **KPNB1** ]  alpha.  Ref: 12504098 Biochem Biophys Res Commun, 2003
